# Supplementary material for: On the resilience of magic number theory for conductance ratios of aromatic molecules
Source: Sci Rep. 2019 Mar 5;9:3478. doi: 10.1038/s41598-019-39937-1 (PMC6401003; doi:10.1038/s41598-019-39937-1)
Supplement: Supplementary file 1 — Supplementary Information [file 41598_2019_39937_MOESM1_ESM.pdf]

Supplementary Information for

On the resilience of magic number theory for conductance ratios of aromatic molecules

Lara Ulčakar[1], Tomaž Rejec[2,1], Jure Kokalj[3,1], Sara Sangtarash*, Hatef Sadeghi*, Anton Ramšak[2,1], John H. Jefferson* and Colin J. Lambert*

* Dept. of Physics, Lancaster University, Lancaster, LA1 4YB, United Kingdom.

[1] J. Stefan Institute, Ljubljana, Slovenia.

[2] Faculty of Mathematics and Physics, University of Ljubljana, Ljubljana, Slovenia.

[3] Faculty of Civil and Geodetic Engineering, University of Ljubljana, Ljubljana, Slovenia.

Supplementary Note 1: The model

The system consists of a molecular core, coupled to conducting leads. Non-interacting part of the core is described by the tight binding model

$$H = -\sum_{i,j,s} \gamma_{ij} c^\dagger_{i,s} c_{j,s} + \sum_i \varepsilon_i n_i. \tag{1}$$

Here i and j run over the sites of the molecule, i.e. the p_z orbitals centred on each of the carbon atoms. $c^\dagger_{i,s}$ and $c_{i,s}$ are the electron creation and annihilation operators for the orbital centred on site i and with spin s. $n_{i,s} = c^\dagger_{i,s} c_{i,s}$ is the electron number operator with $n_i = \sum_s n_{i,s}$. ε_i is the energy of the orbital relative to that at the chiral symmetric point. γ_{ij} are hopping integrals. Taking into account that the next nearest neighbour hoppings in graphene are at least an order of magnitude smaller than the nearest neighbour ones [3], in what follows we retain only the nearest neighbour hopping integrals that we set to $\gamma = 2.4$eV.

When the Coulomb electron-electron interaction is taken into account, we include it according to the Parr-Pariser-Pople (PPP) model [1, 2] and the whole Hamiltonian is of the form

$$H^{int} = H + \sum_i U_{ii}(n_{i,\uparrow} - \frac{1}{2})(n_{i,\downarrow} - \frac{1}{2}) + \frac{1}{2}\sum_{i,j\neq i} U_{ij}(n_i - 1)(n_j - 1). \tag{2}$$

It consists of the on-site interaction U_{ii} and the long range interaction U_{ij}. For the latter we

use the Ohno interpolation [4]

$$U_{ij} = \lambda \begin{cases} U_0, & i = j, \\ U_0 \left[1 + \left(\frac{U_0}{e^2/4\pi\epsilon_0 d_{ij}} \right)^2 \right]^{-1/2}, & i \neq j, \end{cases} \tag{3}$$

where $U = \lambda U_0$ is an interaction strength, $U = U_0 = 11.13$eV giving the physical value of the interaction. d_{ij} is the distance between sites i and j, for nearest-neighbour sites is equal to $d_0 = 1.41$Å.

The leads are modelled as chains of atoms on sites i connected by nearest neighbour hopping integrals γ_0 and are included in the system with a term $-\sum_{i,s,\alpha} \gamma_0 c^\dagger_{\alpha,i+1,s} c_{\alpha,i,s} + \text{H.c.}$. Here $\alpha \in \{L, R\}$ labels the left (source) and the right (drain) lead, respectively, and $i \geq 1$ runs over all atomic sites of one lead. $c^\dagger_{\alpha,i,s}$ and $c_{\alpha,i,s}$ are the electron creation and annihilation operator for lead sites, respectively. Eigenstates of an infinite lead are plane waves with wave vector k and eigenenergy $E(k) = -2\gamma_0 \cos k$. The coupling between lead and the molecule is described by $-\sum_{\alpha,s} V c^\dagger_{\alpha,1,s} c_{i_\alpha,s} + \text{H.c.}$. Here V is the hopping integral between the lead site closest to the molecule and the molecular site i_α to which lead α is attached. We take a wide-band limit, $\gamma_0 = 10\gamma$ and $V = \gamma$. The appropriate quantity that describes strength of coupling between the molecule and the lead is the spectral width

$$\Gamma_{\alpha,ji}(k) = \delta_{ii_\alpha} \delta_{ji_\alpha} \frac{2V^2}{\gamma_0} \sin k. \tag{4}$$

This is the relevant coupling parameter since it includes the density of states of the lead, $\rho \propto 1/\gamma_0$, and the probability for an electron to jump between the lead and the molecule, V^2. It is constant in the energy interval of interest, $\Gamma_\alpha = \frac{2V^2}{\gamma_0} = 0.2\gamma$ and small, justifying the weak coupling limit. The retarded Green's function of the whole system can be expressed in the elastic cotunneling approximation we adopt for the Lanczos calculation which has a simple form

$$\mathcal{G}(E)^{-1} = [G(E)]^{-1} - \Sigma_L - \Sigma_R, \tag{5}$$

where $G(E)$ is the Green's function for the isolated molecule and the influence of the leads is included via Σ_α, the retarded self-energy. Its value due to coupling with lead α is

$$\Sigma_{\alpha,ji}(k) = \delta_{ii_\alpha} \delta_{ji_\alpha} \frac{-V^2}{\gamma_0} e^{ik}. \tag{6}$$

Recently it was shown experimentally [5] that molecular levels shift as a result of electron interaction with image charges in the metal leads, resulting in a HOMO-LUMO gap renormalization. We take the image charge effects into account by analytically solving [6] the Poisson's

equation for the electrostatic Green's function in a simplified geometry, namely we assume the leads are two infinite parallel plates. The renormalized interaction values are

$$U_{ij}^{scr} = U_{ij} + \frac{e^2}{4\pi\epsilon_0}\sum_{\sigma=\pm1}\sum_{n=1}^{\infty}\sigma\left(\frac{1}{\sqrt{(x_i-x_j)^2+(y_i-y_j)^2+[2nL-(z_i-\sigma z_j)]^2}} + \frac{1}{\sqrt{(x_i-x_j)^2+(y_i-y_j)^2+[2nL+(z_i-\sigma z_j)]^2}}\right) - \frac{e^2/4\pi\epsilon_0}{\sqrt{(x_i-x_j)^2+(y_i-y_j)^2+(z_i+z_j)^2}}. \tag{7}$$

Here $\mathbf{r_i} = (x_i, y_i, z_i)$ is a vector pointing to site i and L is the distance between the leads. It depends on the distance between the connectivities and on d - the distance between the lead and the site, which it is connected to. d is measured in units of d_0 - the lattice constant.

Supplementary Note 2: The Hartree-Fock method

The Hartree-Fock method (HF) [7] is an approximation in which the interacting term in the PPP Hamiltonian is evaluated to the first order, leading to an effective HF Hamiltonian

$$H^{HF} = H + \sum_{i,s} U_{ii}(n_{i,s} - \frac{1}{2})\left\langle n_{i,\bar{s}} - \frac{1}{2}\right\rangle + \sum_{j\neq i} U_{ij}(n_i - 1)\langle n_j - 1\rangle - \sum_{j\neq i,s} U_{ij} c_{i,s}^{\dagger} c_{j,s}\left\langle c_{j,s}^{\dagger} c_{i,s}\right\rangle. \tag{8}$$

$\bar{s}$ denotes anti-parallel spin polarization of s. In the restricted Hartree-Fock (HF) approximation the first two terms are zero because $\langle n_{i,\uparrow}\rangle = \langle n_{i,\downarrow}\rangle = \frac{1}{2}$ which is due to chiral symmetry that is not broken in the HF approximation (see Supplementary Note 3). The Hamiltonian in the HF approximation simplifies to the tight binding Hamiltonian with effective hoppings

$$\gamma_{ij}^{HF} = \gamma_{ij} + U_{ij}\left\langle c_{j,s}^{\dagger} c_{i,s}\right\rangle. \tag{9}$$

New long-range hoppings are introduced for i and j on different sublattices. The conductance can again be calculated with the Landauer-Büttiker formula [8, 9]

$$\sigma = \frac{2e^2}{h}T(0),$$

where $T(0)$ is the transmitivity at the gap centre $E_F = 0$ as calculated from the HF Hamiltonian.

Supplementary Note 3: Hartree-Fock approximation preserves chiral symmetry

For bipartite lattice models the chiral symmetry [17] is defined with the operator $\mathcal{S}$ which acts on a site creation and annihilation operator as

$$\mathcal{S}c^{\dagger}_{i_\alpha,s}\mathcal{S}^{-1} = (-1)^{\alpha}c_{i_\alpha,s}, \qquad \mathcal{S}c_{i_\alpha,s}\mathcal{S}^{-1} = (-1)^{\alpha}c^{\dagger}_{i_\alpha,s}, \qquad \mathcal{S}i\mathcal{S}^{-1} = -i, \tag{10}$$

where $\alpha \in \{0,1\}$ is the sublattice index. A system has a symmetry whenever the equality $\mathcal{S}H\mathcal{S}^{-1} = H$ is satisfied, H being the system Hamiltonian. In the cases of tight binding and PPP model this is true when hopping amplitudes γ_{ij} between sites from the same sublattice (same sublattice index) and on-site energies ε_i are zero. Systems with broken chiral symmetry are qualitatively different from systems with chiral symmetry, which is why the PPP model is a meaningful interaction expansion of the original tight binding model. An important criterion as to whether Hartree-Fock is a good approximation is whether it conserves chiral symmetry and is so qualitatively equal to the original PPP and tight binding model. This is indeed the case, as we show below.

The HF approximation transforms the PPP model to the tight binding model with long range hopping amplitudes (9) and the chiral symmetry is therefore preserved if $\langle c^{\dagger}_{i_\alpha,s}c_{j_\alpha,s}\rangle = 0$. The following calculation proves that HF preserves chiral symmetry: The HF Hamiltonian is calculated iteratively and self-consistently and the first iteration starts with the tight binding Hamiltonian, which is chiral symmetric because on the leads and on the molecule only nearest neighbour sites are connected. If one proves that $\langle c^{\dagger}_{i_\alpha,s}c_{j_\alpha,s}\rangle = 0$ for a chiral symmetric system, then after the first iteration no inter-lattice hoppings are introduced and the Hamiltonian remains chiral symmetric with $\langle c^{\dagger}_{i_\alpha,s}c_{j_\alpha,s}\rangle = 0$. The system therefore stays chiral symmetric for all later iterations proving that the HF Hamiltonian is chiral symmetric. Single particle tight binding Hamiltonian of the whole system has the block off-diagonal form

$$H = \begin{pmatrix} 0 & C \\ C^{\dagger} & 0 \end{pmatrix}, \tag{11}$$

where the connectivity matrix C contains hopping amplitudes γ_{ij} between sites on different sublattices. A non-interacting system has chiral symmetry if there exists an unitary operator U_S that transforms the Hamiltonian as $U_S^{\dagger}HU_S = -H$ [17]. For this system the corresponding symmetry operator is the Pauli operator s_z acting on different sublattice spaces. The single

particle eigenstate $|\psi_{k,s}\rangle$ with energy eigenvalue E_k can be decomposed into two components, one on sublattice $\alpha = 0$ and another on $\alpha = 1$,

$$|\psi_{k,s}\rangle = |\psi^0_{k,s}\rangle|\alpha = 0\rangle + |\psi^1_{k,s}\rangle|\alpha = 1\rangle, \qquad |\psi^\alpha_{k,s}\rangle = \sum_i \psi_{k,i_\alpha} c^\dagger_{i_\alpha,s}|0\rangle. \tag{12}$$

where $\psi_{k,i,s} = \psi_{k,i,-s} = \psi_{k,i}$ and $|\alpha\rangle$ represents a state on the sublattice with index α and $|0\rangle$ the vacuum state. In chiral symmetric systems, every $|\psi_{k(E),s}\rangle$ with eigenenergy E has a partner $s_z|\psi_{k(E),s}\rangle = |\psi_{k(-E),s}\rangle$ with eigenvalue $-E$ and eigenvector

$$|\psi_{k(-E),s}\rangle = |\psi^0_{k(E),s}\rangle|\alpha = 0\rangle - |\psi^1_{k(E),s}\rangle|\alpha = 1\rangle. \tag{13}$$

Here we assumed the there is a HOMO-LUMO gap for a half-filled system, so there is no state at $E_F = 0$. The expectation value $\langle c^\dagger_{i_\alpha,s} c_{j_\alpha,s}\rangle$ is calculated for a half-filled system in the ground state of the HF Hamiltonian, that has all of the eigenstates with $E_k < E_F$ filled with electrons up to Fermi level $E_F = 0$,

$$\langle c^\dagger_{i_\alpha,s} c_{j_\alpha,s}\rangle = \sum_{k,E_k<0} \psi_{k,i_\alpha}\psi^*_{k,j_\alpha}. \tag{14}$$

For a completely full system this expectation value is zero i.e.,

$$\sum_{k,E_k<0} \psi_{k,i_\alpha}\psi^*_{k,j_\alpha} + \sum_{k,E_k>0} \psi_{k,i_\alpha}\psi^*_{k,j_\alpha} = 2\langle c^\dagger_{i_\alpha,s} c_{j_\alpha,s}\rangle = 0. \tag{15}$$

Here we used $\psi_{k(E),i_\alpha}\psi^*_{k(E),j_\alpha} = \psi_{k(-E),i_\alpha}\psi^*_{k(-E),j_\alpha}$ that is evident from equation (13). This proves that $\langle c^\dagger_{i_\alpha,s} c_{j_\alpha,s}\rangle = 0$ for a chiral symmetric Hamiltonian, which further proves that HF Hamiltonian stays chiral symmetric. From here also follows that the single-particle HF Hamiltonian is again of off-diagonal block form as the non-interacting one in equation (11)

$$H^{HF} = \begin{pmatrix} 0 & C^{HF} \\ C^{HF\,\dagger} & 0 \end{pmatrix}, \tag{16}$$

where C^{HF} is the Hartree-Fock connectivity matrix filled with effective hopping amplitudes from equation (9), $C^{HF}_{ij} = \gamma^{HF}_{ij}$.

Supplementary Note 4: Validity of the Hartree-Fock method

Lanczos diagonalization produces exact results in the limit of weak coupling between the molecule and metallic leads but processing time grows exponentially with number of

atomic sites making the calculation impractical for larger molecules. Calculation with the Hartree-Fock method is much faster, growing polynomially with size. Although it is an approximate mean-field method and is known to accurately describe systems with sufficiently weak Coulomb interactions. It is therefore prudent to test its validity for the interacting PPP model of polyaromatic molecules, for which the Coulomb interaction might be significant. Hartree-Fock results for transmitivity, density of states and energy gap are compared with results from the Lanczos method. Fig. 3(b), Fig. (4)b from the main text and Supplementary Fig. 7(b), Supplementary Fig. 8(b) and Supplementary Fig. 9(b) show good agreement of energy gaps for different molecules according to HF and Lanczos method for interaction strength up to $\lambda = 1.5$.

Supplementary Fig. 13 shows comparison between transmitivity of energy from HF and Lanczos calculation for various interaction strengths with no screening and Supplementary Fig. 14 with screening for different lead distances d. Graphs show good agreement between methods for interaction strength lower than $\lambda = 1.5$ for energies in the HOMO-LUMO gap and even for low lying excited states above the gap.

The reason for deviations of Hartree-Fock calculations from Lanczos' at $\lambda \approx 1.5$ can be explained by the emergence of an antiferromagnetic Hartree-Fock ground state. This can be seen by using the unrestricted Hartree-Fock approximation, which does not enforce the constraint that the expected number of electrons with spins up is equal to number of electrons with spin down. Staggered magnetization is defined as $\langle n_{i,\uparrow}\rangle - \langle n_{i,\downarrow}\rangle$. Its dependence on interaction strength is plotted for various molecules in Supplementary Fig. 15. It is evident that staggered magnetization becomes non-zero at around $\lambda \approx 1.5$. This phenomena occurs also in graphene when described by the Hartree-Fock PPP model [7]. Phase transition happens at slightly lower λ than for anthanthrene, which is consistent with the observation that the larger the molecule is the lower the phase transition point below which the system is paramagnetic.

Supplementary Note 5: Infinite range interaction limit

The PPP model can be considered as a model that is intermediate between two extreme cases, a system with localized interaction and a system with infinite range interaction. The first one is described by the Hubbard model that has $U_{ii} = U$ and $U_{i,j\neq i} = 0$. As seen from equation (8) in the HF approximation of the Hubbard model the interaction has no effect. The model with infinite range interaction has $U_{ij} = \tilde{U}$ for all pairs of i and j where $\tilde{U}$ is the

average value of the PPP interaction integrals in a given molecule. In this section the M-table in the infinite range interaction limit is derived. Since the PPP interaction is somewhere in between both limiting cases (Hubbard and infinite-range), its M-table will be somewhere in between M-tables of those two limit cases.

The non-interacting Hamiltonian H of an isolated molecule, that is a bipartite lattice with $\frac{N}{2}$ sites per sublattice, can be expressed in terms of a $\frac{N}{2} \times \frac{N}{2}$ connectivity matrix C as in equation (11) and is symmetric under the chiral symmetry operator s_z. When wave functions are expressed in terms of wave functions on each sublattice, see equation (12), the Schrödinger equation is

$$\begin{aligned} C|\psi^1_{k,s}\rangle &= E_k|\psi^0_{k,s}\rangle, \\ C^\dagger|\psi^0_{k,s}\rangle &= E_k|\psi^1_{k,s}\rangle. \end{aligned} \tag{17}$$

Since the system has a gap and no gap states, $E_k \neq 0$ and the eigenstate sublattice wave functions are related,

$$|\psi^0_{k,s}\rangle = \frac{C|\psi^1_{k,s}\rangle}{E_k}. \tag{18}$$

Therefore, only the problem on one sublattice needs to be solved,

$$C^\dagger C|\psi^1_{k,s}\rangle = E_k^2|\psi^1_{k,s}\rangle, \tag{19}$$

where $C^\dagger C$ is Hermitian and and its eigenvectors form a unitary matrix,

$$V^{(1)} = \left(|\psi^1_{1,s}\rangle, |\psi^1_{2,s}\rangle, \ldots, |\psi^1_{\frac{N}{2},s}\rangle\right). \tag{20}$$

Since the probabilities to find an electron on each sublattice are equal:

$$\langle\psi^0_{k,s}|\psi^0_{k,s}\rangle = \frac{\langle\psi^1_{k,s}|C^\dagger C|\psi^1_{k,s}\rangle}{E_k^2} = \langle\psi^1_{k,s}|\psi^1_{k,s}\rangle, \tag{21}$$

the normalized eigenstates with $E_k > 0$ ($E_k < 0$) are therefore

$$\begin{aligned} |\psi_{k,s}\rangle &= \pm\frac{1}{|E|}C|\psi^1_{k,s}\rangle|\alpha = 0\rangle + |\psi^1_{k,s}\rangle|\alpha = 1\rangle \\ &= \pm C\left(C^\dagger C\right)^{-\frac{1}{2}}|\psi^1_{k,s}\rangle|\alpha = 0\rangle + |\psi^1_{k,s}\rangle|\alpha = 1\rangle. \end{aligned} \tag{22}$$

Let us form a $N \times \frac{N}{2}$ matrix V containing occupied ($E_k < 0$) eigenstates of H in its columns:

$$V = \frac{1}{\sqrt{2}}\begin{pmatrix} -C\left(C^\dagger C\right)^{-\frac{1}{2}} \\ 1 \end{pmatrix} V^{(1)}. \tag{23}$$

In terms of V the single-particle correlations are

$$\langle c_{is}^{\dagger} c_{js} \rangle = \sum_{E<0} \psi_{ni}^{*} \psi_{nj} = \left(V V^{\dagger} \right)_{ji} \tag{24}$$

with

$$V V^{\dagger} = \begin{pmatrix} \frac{1}{2} & -\frac{1}{2} C \left(C^{\dagger} C \right)^{-\frac{1}{2}} \\ -\frac{1}{2} \left(C^{\dagger} C \right)^{-\frac{1}{2}} C^{\dagger} & \frac{1}{2} \end{pmatrix}. \tag{25}$$

In particular, for j and i in the same sublattice $\langle c_{j,s}^{\dagger} c_{i,s} \rangle = \frac{1}{2} \delta_{ji}$.

For conductance ratios in the limit of weak coupling between the molecule and leads we consider the Green's function at the centre of the HOMO-LUMO gap

$$G(0) = (0 - H)^{-1} = \begin{pmatrix} 0 & \bar{M} \\ \bar{M}^{\dagger} & 0 \end{pmatrix} \tag{26}$$

which is expressed in terms of the M-table which can be calculated from the connectivity matrix as

$$\bar{M} = - \left(C^{\dagger} \right)^{-1}. \tag{27}$$

Note that the M-table as defined here does not contain integers in general, but the non-integer factors are cancelled out when calculating conductance ratios.

The PPP Hamiltonian in the HF approximation leads to an effective Hamiltonian H^{HF} that has chiral symmetry so it can also be expressed in the form of equation (11), that is in terms of the HF connectivity matrix C^{HF} containing effective hoppings. The HF conductance ratios are then given by the HF M-table

$$G^{HF}(0) = \left(0 - H^{HF} \right)^{-1} = \begin{pmatrix} 0 & \bar{M}^{HF} \\ \bar{M}^{HF\,\dagger} & 0 \end{pmatrix}, \tag{28}$$

$$\bar{M}^{HF} = - \left(C^{HF\,\dagger} \right)^{-1}. \tag{29}$$

(In the main text M^{int} is an M table for interaction system for general method of calculation, here we explicitly use the name of HF approximation.) Assuming the interaction is independent of distance (infinite range interaction, $U_{ji} = \tilde{U}$), the HF self-consistency equation reads, see Eqs. (8) and (25):

$$C^{HF} = C + \frac{\tilde{U}}{2} C^{HF} \left(C^{HF\,\dagger} C^{HF} \right)^{-\frac{1}{2}}. \tag{30}$$

Its solution is

$$C^{HF} = C + \frac{\tilde{U}}{2} C \left(C^\dagger C \right)^{-\frac{1}{2}}. \tag{31}$$

Note that the HF Hamiltonian

$$\begin{aligned} C^{HF} |\psi^1_{k,s}\rangle &= \left(E_k + \frac{\tilde{U}}{2} \frac{E_k}{|E_k|} \right) |\psi^0_{k,s}\rangle, \\ C^{HF\,\dagger} |\psi^0_{k,s}\rangle &= \left(E_k + \frac{\tilde{U}}{2} \frac{E_k}{|E_k|} \right) |\psi^1_{k,s}\rangle, \end{aligned} \tag{32}$$

see Eq. (17) and (19), has the same eigenvectors as the non-interacting one, with the HOMO-LUMO gap increased by the value of the interaction $\tilde{U}$ being the only difference in the eigenvalue spectrum. The infinite range HF Hamiltonian can be expressed with non-interacting one as

$$H^{HF} = H + \frac{\tilde{U}}{2} \mathrm{sgn} H. \tag{33}$$

sgn$A$ is a matrix one gets if one diagonalizes the matrix $A = P \Lambda P^{-1}$ and then replaces eigenvalues Λ_{ii} by their sign $[\mathrm{sgn}\Lambda]_{ii} = \Lambda_{ii}/|\Lambda_{ii}|$ and then rotates the matrix back to original basis of A, $\mathrm{sgn}A = P^{-1} \mathrm{sgn}\Lambda P$. Equation (33) follows from the fact that H^{HF} has the off-diagonal form as in (11) and the fact that $E_k/|E_k|$ are eigenvalues of sgn$H$.

The exact PPP Hamiltonian in the infinite range interaction limit can be written as

$$H^{int} = H + \frac{\tilde{U}}{2} (n - N)^2 - \frac{\tilde{U}}{4} N, \tag{34}$$

with n being the total number of electrons, $n = \sum_i n_i$. Again, for each fixed n Slater determinants built from single electron eigenstates of the non-interacting Hamiltonian H are the many-body eigenstates of H^{int}. There is additional dependence of many-body eigenenergies on n though, leading to an increase of the HOMO-LUMO gap by $\tilde{U}$, as in the HF approximation. Both the exact and the HF Green's functions are therefore the same in the infinite range interaction limit for a half filled system. From here follows that the HF results for long range interaction are exact ones and not only an approximation. We denote the exact long range results as $X^{HF} \to \tilde{X}$.

For such an interaction the HF M-table is related to the non-interacting M-table, Eqs. (29) and (31)

$$\tilde{\bar{M}} = \bar{M} \left(1 + \frac{\tilde{U}}{2} \left(C^\dagger C \right)^{-\frac{1}{2}} \right)^{-1} \tag{35}$$

The exact expression for Green's function of the core for the infinite range interaction is (see Eq. (33))

$$\tilde{G}(0) = -(H + \frac{1}{2}\tilde{U}\mathrm{sgn}H)^{-1}. \tag{36}$$

For $\tilde{U} \gg \gamma$ the M-table ratios become independent of the interaction strength and the HF M-table can be rescaled to

$$\frac{\tilde{U}}{4}\tilde{\bar{M}} = \bar{M}\frac{1}{2}\left(C^{\dagger}C\right)^{\frac{1}{2}} = -\frac{1}{2}C\left(C^{\dagger}C\right)^{-\frac{1}{2}} \tag{37}$$

or, using Eq. (25)

$$\frac{\tilde{U}}{4}\tilde{\bar{M}}_{ji} = \langle c_{i,s}^{\dagger}c_{j,s}\rangle. \tag{38}$$

In this limit, all occupied states merge into the HOMO level at $E = -\frac{\tilde{U}}{2}$ and all the empty states merge into the LUMO level at $E = \frac{U}{2}$.

In order to compare the results obtained using infinite range interaction and the PPP parametrisation we show, in Supplementary Fig. 16(a), correlations of the Hartree-Fock conductance ratio for a particular pair of connectivities (horizontal axis) with the non-interacting (blue dots) and the infinite range interaction (orange dots) conductance ratio for the same pair of connectivities. Results for all possible pairs of connectivities are shown. The first observation is in remarkable agreement between infinite range and the PPP model results. In is also clearly demonstrated that non-interacting results (i.e., magic ratios) are correlated with the PPP results on average, but exhibiting noticeable deviations in some cases. Maximum deviations of PPP results compared to non-interaction ratios are limited within the scaling range $0.1 \sim 10$. In Supplementary Fig. 16(b) the same type of analysis is shown but in the limit of large interaction strength, $U \to \infty$. Here the correlation between infinite range and PPP model is more dispersed, but still remarkable.

In Supplementary Fig. 16(c) are shown correlations of the Hartree-Fock conductance ratio (horizontal axis) for a particular pair of connectivities and the infinite-range interaction (orange dots) conductance ratio for the same pair of connectivities and both normalised to the corresponding non-interacting result for $U = \tilde{U}$. From this figure the scaling range $0.1 \sim 10$ of deviations of PPP results compared to non-interaction ratios in even more evident. Also it shows strong correlation of infinite range and the PPP results. Blue dots represent results for the nearest neighbour connectivities only. In Supplementary Fig. 16(d) are shown the results for $U \to \infty$, also in this limit demonstrating robust agreement in infinite range model and the PPP results.

In Supplementary Fig. 17 are presented pyrene results corresponding to Supplementary Fig. 16. Qualitatively all conclusions are unchanged.

Supplementary Note 6: Systematic view on the change of conductance ratios

By comparing magic ratios with conductance ratios for a system with Coulomb interaction, with or without screening, one can analyse the changes and try to find a general description for deviations from magic ratios.

Supplementary Note 6.1: Effects of interaction with no screening

For systems with Coulomb interaction and no screening there is a qualitative rule which describes deviations of conductance ratios from magic ratios. To illustrate this correlation graphs for anthanthrene are shown in Supplementary Fig. 18. Correlation between two quantities is defined as

$$\mathrm{Corr}(x, y) = \frac{1}{N-1} \sum_{i=1}^{N} \left(\frac{x_i - \bar{x}}{\sigma_x} \right) \left(\frac{y_i - \bar{y}}{\sigma_y} \right), \tag{39}$$

with $\bar{x}$ and $\bar{y}$ being the mean values, σ_x and σ_y standard deviations and N the number of samples. In Supplementary Table 1 correlation coefficients are presented. By observing the graph of correlation between the absolute value of elements M-table, $|M_{ij}|$, and the distance between sites i and j, there is no discernible correlation in the non-interacting model case whereas in the interacting model case (see $|M^{HF}|$) there is a negative correlation. It can be therefore expected that the $|M_{ij}|$ number increases from the magic values if the connectivity sites i and j are near and decreases if the connectivity sites i and j are far apart.

This behaviour can be explained by observing the molecule energy spectra at different interaction strengths, shown in Supplementary Fig. 19 and Supplementary Fig. 20. In the limit of strong interaction, energy levels above and below the HOMO-LUMO gap come much closer to each other, as if they were squeezed to a very narrow band. Energy levels are renormalized so the HOMO-LUMO gap stays constant with interaction. By definition, elements of the M-table are not affected by such renormalization factors. In the limit of strong interaction one can therefore make an approximation for the eigenenergy E_k of the eigenstate $|\psi_{k,s}\rangle$: $E_k \approx E^{HF}_{HOMO}$ if $E_k < E_F$ and $E_k \approx -E^{HF}_{HOMO}$ if $E_k > E_F$, E^{HF}_{HOMO} being the highest occupied energy level and E_F the Fermi energy. This approximation for eigenenergies is exact in the limit of infinite range interaction of infinite strength as shown in Supplementary Note

5. The ij-th element of the Green's function of the isolated molecule can be approximated with

$$G_{ij}^{HF}(0) = \sum_{k,s} -\frac{1}{E_k}\psi_{k,i}\psi_{k,j}^* \approx -\frac{2}{E_{HOMO}^{HF}}\sum_s \langle c_{i,s}^\dagger c_{j,s}\rangle^{HF}, \tag{40}$$

where $\psi_{k,i,s} = \psi_{k,i,-s} = \psi_{k,i}$ are wave function coefficients of the eigenstates of HF Hamiltonian. The expectation value $\langle c_{i,s}^\dagger c_{j,s}\rangle^{HF}$ is calculated for a ground state of the interacting Hamiltonian, namely the ground state of HF Hamiltonian when working with the HF method. As shown in Supplementary Table 1, Hartree-Fock $\langle c_{i,s}^\dagger c_{j,s}\rangle^{HF}$ is perfectly correlated with non-interacting $\langle c_{i,s}^\dagger c_{j,s}\rangle$, which suggests that wave functions do not change with interaction in HF method. Also, in Supplementary Fig. 19 and Supplementary Fig. 20 the $\psi_{k,i}\psi_{k,j}^*$ are presented at every level k at different U and it is evident that this quantity, directly connected to wave functions, does not change with interaction. The same observation is analytically derived in Supplementary Note 5 for a model with infinite range interaction, from which its follows that this expectation values can be evaluated in the non-interacting ground state. In the limit of strong interaction one can therefore qualitatively estimate conduction ratios according to a simple non-interacting expression

$$\frac{\sigma_{ij}^{HF}}{\sigma_{kl}^{HF}} = \left[\frac{M_{ij}^{HF}}{M_{kl}^{HF}}\right]^2 \approx \left|\frac{\sum_s \langle c_{i,s}^\dagger c_{j,s}\rangle}{\sum_s \langle c_{k,s}^\dagger c_{l,s}\rangle}\right|^2. \tag{41}$$

As seen from equation (38), the approximation becomes exact for the infinite range interaction model with infinite interaction strength. The fact that above relation qualitatively reproduces conductance ratios can be read from Table 1 in the main text. M_{ij}^{HF} are therefore proportional to $\sum_s \langle c_{i,s}^\dagger c_{j,s}\rangle$ and since it is known [16] that this quantity decreases with distance between site i and site j this explains why M_{ij}^{HF} also decreases from magic integers if i and j are far apart.

Supplementary Note 6.2: Effects of lead screening

In some cases screening does not affect the conductance ratios whilst in others they are changed drastically. As its name implies screening usually decreases effective interaction strength. However, the width of the HOMO-LUMO gap is changed differently for different lead connectivities because screening depends on the distance between the two leads and the distance depends on connectivity. The consequence is that the calculated M-tables are

different for different connectivities, for example $[M_{1,2}^{HF}]_{ij} \neq [M_{3,7}^{HF}]_{ij}$, in first case the leads are connected to sites 1 and 2 and in second case to 3 and 7.

Change of conductance ratios can be seen as a combined effect of different HOMO-LUMO gap renormalization and different M-tables. This is evident by rewriting conductance ratios in the basis of energy eigenstates $|\psi_{k,s}\rangle_{ij}$ and $|\psi_{k,s}\rangle_{lm}$, which are different for different connectivities. Eigenenergies E_k^{ij} and coefficients $\psi_{k,i}^{ij}$ correspond to the system with connectivity $i-j$ while E_k^{lm} and $\psi_{k,l}^{lm}$ correspond to the system with connectivity $l-m$.

$$\frac{\sigma_{ij}^{HF}}{\sigma_{lm}^{HF}} = \left|\frac{G_{ij}^{HF}(0)}{G_{lm}^{HF}(0)}\right|^2 = \left|\frac{\sum_{k,s} -\frac{1}{E_k^{ij}}(\psi_{k,j}^{ij})^*\psi_{k,i}^{ij}}{\sum_{k,s} -\frac{1}{E_k^{lm}}(\psi_{k,m}^{lm})^*\psi_{k,l}^{lm}}\right|^2 = \left(\frac{E_g^{lm}}{E_g^{ij}}\right)^2 \left|\frac{[M_{ij}^{HF}]_{ij}}{[M_{lm}^{HF}]_{lm}}\right|^2, \tag{42}$$

where E_g^{ij} is the HOMO-LUMO gap of a system with connectivity $i-j$ and M-table ratio in case of screening as

$$\frac{[M_{ij}^{HF}]_{ij}}{[M_{lm}^{HF}]_{lm}} = \frac{\sum_{k,s} -\frac{E_g^{ij}}{E_k^{ij}}(\psi_{k,j}^{ij})^*\psi_{k,i}^{ij}}{\sum_{k,s} -\frac{E_g^{lm}}{E_k^{lm}}(\psi_{k,m}^{lm})^*\psi_{k,l}^{lm}}. \tag{43}$$

In Supplementary table 2 are shown conductance ratios in case of no screening, screening, M-table ratio squared and gap ratio squared for different connectivities. From it can be seen that the conductance ratio in case of screening can be expressed as a product of gap ratio squared and M-table ratio squared. Small deviations of the product from conductance ratios are due to coupling to leads. This suggests that consequences of screening can be interpreted as a combination of both mechanisms.

Supplementary Note 7: Electron currents

An illustrative representation of conduction processes is to plot electron currents through the molecule in terms of so called bond currents [14]. The bond current I_{ij} is defined as the current that flows along a bond connecting site i and site j. At $T = 0\,\mathrm{K}$ and infinitesimal difference of lead chemical potentials $\mu_L - \mu_R = eV$ and $V \to 0$, it is equal to the expectation value of j_{ij}, the current operator between sites i and j

$$j_{ij} = i\sum_s (\gamma_{ij} c_{i,s}^\dagger c_{j,s} - \gamma_{ij}^* c_{j,s}^\dagger c_{i,s}), \tag{44}$$

in the single-particle scattering state at the Fermi energy from left (source) lead state $|\varphi_{k_F,s,L}\rangle =$

$\sum_j \varphi_{k_F,j} c^\dagger_{j,s,L}|0\rangle$. The final expression that was used to calculate bond currents is

$$\frac{I_{ij}}{V} = \frac{e^2}{h}\frac{1}{\gamma_0}\mathrm{Im}\left[\varphi^*_{k_F,i}(-\gamma_{ij})\varphi_{k_F,j}\right]. \tag{45}$$

Currents can be expressed in terms of Green's function for a real Hamiltonian as

$$\frac{I_{ij}}{V} = \frac{2e^2}{h}\gamma_{ij}(G_{ii_R}(0)\Gamma_{i_R}G_{i_Ri_L}(0)\Gamma_{i_L}G_{i_Lj}(0) - G_{ii_L}(0)\Gamma_{i_L}G_{i_Li_R}(0)\Gamma_{i_R}G_{i_Rj}(0)), \tag{46}$$

while the total current through the junction is equal to

$$\frac{I}{V} = \frac{2e^2}{h}G_{i_Ri_L}(0)\Gamma_{i_L}G_{i_Li_R}(0)\Gamma_{i_R}. \tag{47}$$

The current distribution through the bonds is

$$\frac{I_{ij}}{I} = \gamma_{ij}\frac{G_{ii_R}(0)G_{i_Lj}(0) - G_{ii_L}(0)G_{i_Rj}(0)}{G_{i_Ri_L}(0)}. \tag{48}$$

In Supplementary Fig. 21(b) and (c) , bond currents I_{ij} are plotted as arrows between sites. The arrow direction is that of the current and its thickness the magnitude of the bond current. In the non-interacting case, bond currents flow only along nearest neighbours in contrast to the interacting case, where all atoms from different sublattices become connected in the HF method and (much weaker) currents flow also between non-neighbouring atoms. The HF method does not induce new bond currents between sites of the same sublattice because it does not induce new hoppings γ_{ij}^{HF} between sites within the same sublattice.

A more representative way of showing the currents in the molecule is by site currents, which are defined as a vector sum of bond currents, flowing to and from particular site. An example is shown in Supplementary Fig. 21(d) .

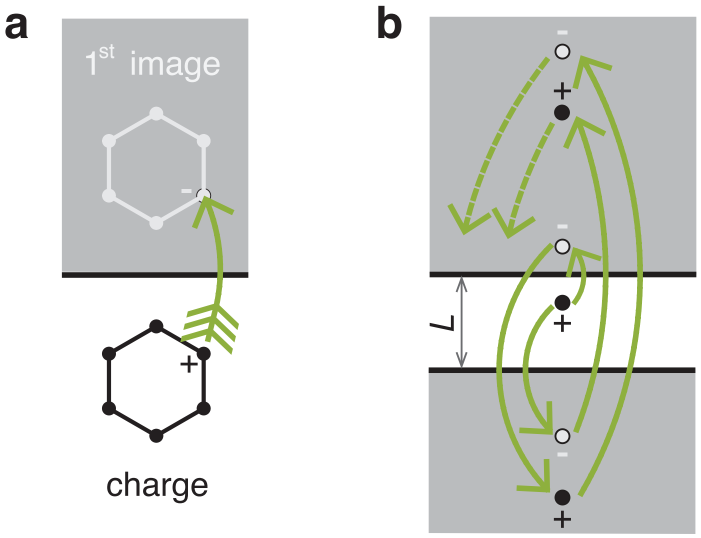

Supplementary Figure 1: Image charges in leads
(a) Charge image of benzene when one lead (grey area) is present. Black and white dots represent positive and negative charges, respectively. Green arrow points to image charge of a particular original charge. (b) A system with two leads separated by a distance L. Two leads act as two parallel mirrors, producing infinite images.

a

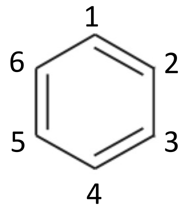

b

C	2	4	6
1	-1	0	-1
3	-1	-1	0
5	0	-1	-1

c

$\bar{M}$	1	3	5
2	-1	-1	1
4	1	-1	-1
6	-1	1	-1

d

$\bar{M}^{int}$	1	3	5
2	-1.049	-1.049	0.884
4	0.884	-1.049	-1.049
6	-1.049	0.884	-1.049

Supplementary Figure 2: M and C tables for benzene
Similar to Fig. 2 in main paper: (a) The benzene core numbering system. (b) The connectivity table C. (c) The non-interacting magic number table $\bar{M}$ corresponding to the benzene lattice. (d) The interacting magic number table $\bar{M}$ calculated with HF approximation corresponding to the benzene lattice.

a

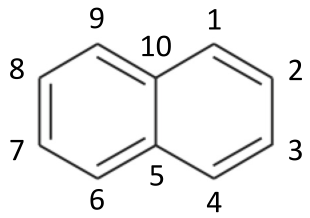

b

C	2	4	6	8	10
**1**	-1	0	0	0	-1
**3**	-1	-1	0	0	0
**5**	0	-1	-1	0	-1
**7**	0	0	-1	-1	0
**9**	0	0	0	-1	-1

c

$\bar{M}$	2	4	6	8	10
**1**	-2	-1	1	-1	1
**3**	2	-2	-1	1	-1
**5**	-1	1	-1	-2	2
**7**	1	-1	1	-1	-2
**9**	-1	1	-1	1	-1

d

$\bar{M}^{int}$	2	4	6	8	10
**1**	-2.078	-1.270	1.051	-0.774	0.732
**3**	1.626	-2.078	-1.167	0.732	-0.611
**5**	-0.611	0.732	-1.167	-2.078	1.626
**7**	0.732	-0.774	1.051	-1.270	-2.078
**9**	-1.167	1.051	-1.363	1.051	-1.167

Supplementary Figure 3: M and C tables for naphthalene
Similar to Fig. 2 in main paper: (a) The naphthalene core numbering system. (b) The connectivity table C. (c) The non-interacting magic number table $\bar{M}$ corresponding to the naphthalene lattice. (d) The interacting magic number table $\bar{M}$ calculated with HF approximation corresponding to the naphthalene lattice.

a

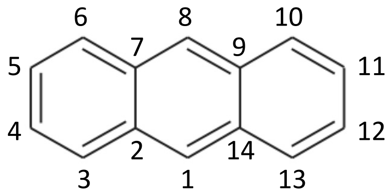

b

C	2	4	6	8	10	12	14
1	-1	0	0	0	0	0	-1
3	-1	-1	0	0	0	0	0
5	0	-1	-1	0	0	0	0
7	-1	0	-1	-1	0	0	0
9	0	0	0	-1	-1	0	-1
11	0	0	0	0	-1	-1	0
13	0	0	0	0	0	-1	-1

c

$\bar{M}$	1	3	5	7	9	11	13
2	-2	-1	1	-1	1	-1	1
4	2	-3	-1	1	-1	1	-1
6	-2	3	-3	-1	1	-1	1
8	4	-2	2	-2	-2	2	-2
10	-2	1	-1	1	-1	-3	3
12	2	-1	1	-1	1	-1	-3
14	-2	1	-1	1	-1	1	-1

d

$\bar{M}^{int}$	1	3	5	7	9	11	13
2	-2.316	-1.436	1.281	-1.697	1.296	-0.921	0.827
4	1.454	-3.264	-1.628	1.281	-0.921	0.648	-0.555
6	-1.153	2.480	-3.264	-1.436	0.827	-0.555	0.446
8	2.987	-1.153	1.454	-2.316	-2.316	1.454	-1.153
10	-1.153	0.446	-0.555	0.827	-1.436	-3.264	2.480
12	1.454	-0.555	0.648	-0.921	1.281	-1.628	-3.264
14	-2.316	0.827	-0.921	1.296	-1.697	1.281	-1.436

Supplementary Figure 4: M and C tables for anthracene

Similar to Fig. 2 in main paper: (a) The anthracene core numbering system. (b) The connectivity table C. (c) The non-interacting magic number table $\bar{M}$ corresponding to the anthracene lattice. (d) The interacting magic number table $\bar{M}$ calculated with HF approximation corresponding to the anthracene lattice.

a

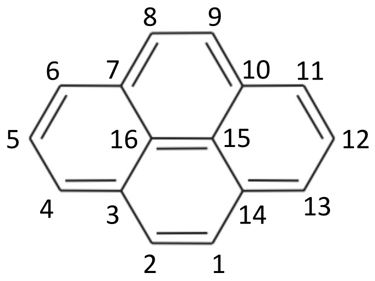

b

C	2	4	6	8	10	12	14	16
1	-1	0	0	0	0	0	-1	0
3	-1	-1	0	0	0	0	0	-1
5	0	-1	-1	0	0	0	0	0
7	0	0	-1	-1	0	0	0	-1
9	0	0	0	-1	-1	0	0	0
11	0	0	0	0	-1	-1	0	0
13	0	0	0	0	0	-1	-1	0
15	0	0	0	0	-1	0	-1	-1

c

$\bar{M}$	1	3	5	7	9	11	13	15
2	-5	-1	1	-1	1	-3	3	2
4	3	-3	-3	3	-3	3	-3	0
6	-3	3	-3	-3	3	-3	3	0
8	1	-1	1	-1	-5	3	-3	2
10	-1	1	-1	1	-1	-3	3	-2
12	1	-1	1	-1	1	-3	-3	2
14	-1	1	-1	1	-1	3	-3	-2
16	2	-2	2	-2	2	0	0	-2

d

$\bar{M}^{int}$	1	3	5	7	9	11	13	15
2	-5.156	-1.522	0.869	-0.733	0.529	-2.019	2.150	1.927
4	2.150	-3.364	-3.563	2.892	-2.019	1.646	-1.674	0.015
6	-2.019	2.892	-3.563	-3.364	2.150	-1.674	1.646	0.015
8	0.529	-0.733	0.869	-1.522	-5.156	2.150	-2.019	1.927
10	-0.733	1.002	-0.958	1.228	-1.522	-3.364	2.892	-2.737
12	0.869	-0.958	0.838	-0.958	0.869	-3.563	-3.563	2.273
14	-1.522	1.228	-0.958	1.002	-0.733	2.892	-3.364	-2.737
16	1.927	-2.737	2.273	-2.737	1.927	0.015	0.015	-2.189

Supplementary Figure 5: M and C tables for pyrene

Similar to Fig. 2 in main paper: (a) The pyrene core numbering system. (b) The connectivity table C. (c) The non-interacting magic number table $\bar{M}$ corresponding to the pyrene lattice. (d) The interacting magic number table $\bar{M}$ calculated with HF approximation corresponding to the pyrene lattice.

a

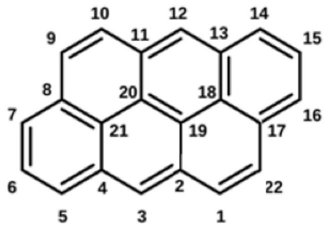

b

C	2	4	6	8	10	12	14	16	18	20	22
1	-1	0	0	0	0	0	0	0	0	0	-1
3	-1	-1	0	0	0	0	0	0	0	0	0
5	0	-1	-1	0	0	0	0	0	0	0	0
7	0	0	-1	-1	0	0	0	0	0	0	0
9	0	0	0	-1	-1	0	0	0	0	0	0
11	0	0	0	0	-1	-1	0	0	0	-1	0
13	0	0	0	0	0	-1	-1	0	-1	0	0
15	0	0	0	0	0	0	-1	-1	0	0	0
17	0	0	0	0	0	0	0	-1	-1	0	-1
19	-1	0	0	0	0	0	0	0	-1	-1	0
21	0	-1	0	-1	0	0	0	0	0	-1	0

c

$\bar{M}$	1	3	5	7	9	11	13	15	17	19	21
2	-1	-7	4	-4	1	-1	1	-1	1	-2	3
4	1	-3	-4	4	-1	1	-1	1	-1	2	-3
6	-1	3	-6	-4	1	-1	1	-1	1	-2	3
8	1	-3	6	-6	-1	1	-1	1	-1	2	-3
10	-1	3	-6	6	-9	-1	1	-1	1	-2	3
12	3	-9	8	-8	7	-7	-3	3	-3	6	1
14	-6	8	-6	6	-4	4	-4	-6	6	-2	-2
16	6	-8	6	-6	4	-4	4	-4	-6	2	2
18	3	1	-2	2	-3	3	-3	3	-3	-4	1
20	-2	6	-2	2	2	-2	2	-2	2	-4	-4
22	-9	7	-4	4	-1	1	-1	1	-1	2	-3

d

$\bar{M}^{int}$	1	3	5	7	9	11	13	15	17	19	21
2	-2.246	-8.091	3.345	-3.346	0.637	-0.920	1.274	-1.218	1.615	-3.604	3.596
4	1.026	-4.212	-5.330	4.696	-0.898	1.274	-1.276	1.064	-1.164	2.822	-4.910
6	-0.656	2.538	-7.386	-5.852	1.161	-1.218	1.064	-0.837	0.841	-2.099	3.933
8	0.518	-2.080	5.866	-7.028	-2.298	1.615	-1.164	0.841	-0.765	2.050	-4.638
10	-0.355	1.423	-4.005	4.473	-9.974	-2.246	1.026	-0.656	0.518	-1.391	3.182
12	1.423	-5.436	4.197	-4.523	5.152	-8.091	-4.212	2.538	-2.080	5.515	0.883
14	-4.005	4.197	-2.595	2.751	-2.244	3.345	-5.330	-7.386	5.866	-1.294	-1.323
16	4.473	-4.523	2.751	-2.880	2.286	-3.346	4.696	-5.852	-7.028	1.439	1.397
18	3.182	0.883	-1.323	1.397	-2.441	3.596	-4.910	3.933	-4.638	-4.751	0.654
20	-1.391	5.515	-1.294	1.439	2.484	-3.604	2.822	-2.099	2.050	-5.732	-4.751
22	-9.974	5.152	-2.244	2.286	-0.443	0.637	-0.898	1.161	-2.298	2.484	-2.441

Supplementary Figure 6: M and C tables for anthanthrene
Similar to Fig. 2 in main paper: (a) The anthanthrene core numbering system. (b) The connectivity table C. (c) The non-interacting magic number table $\bar{M}$ corresponding to the anthanthrene lattice. (d) The interacting magic number table $\bar{M}$ calculated with HF approximation corresponding to the anthanthrene lattice.

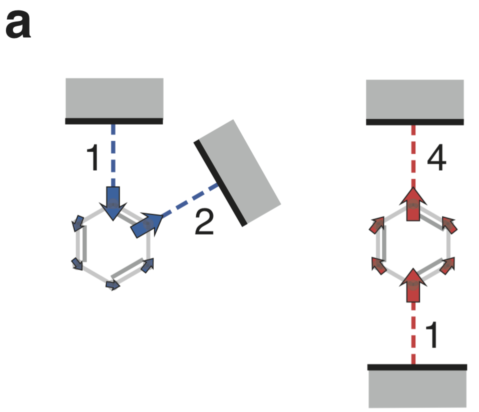

b

λ	ratio HF	ratio Lanczos	gap (eV) HF	gap (eV) Lanczos
0	1.00	1.00	4.80	4.77
0.5	1.24	1.30	8.02	8.03
**1**	**1.39**	**1.60**	**11.27**	**11.34**
1.5	1.50	1.98	14.53	14.84
2	1.58	2.52	17.80	18.65

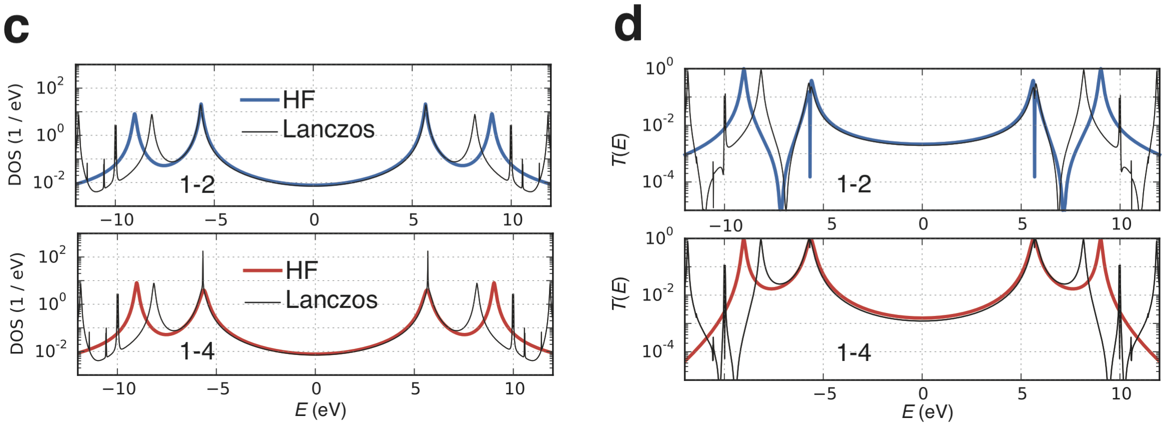

Supplementary Figure 7: results for benzene

(a) Two examples of benzene molecule with different connectivities ($1-2$ and $1-4$) to leads (grey area). Arrows represent electron site current. (b) A table of conduction ratios and HOMO-LUMO gap according to HF and Lanczos calculation for interacting case with interaction strength $\lambda = U/U_0$ with no screening. (c) Lanczos (black) and HF (red, blue) density of states as a function of energy at two different connectivities at $U = U_0$ and $d = d_0$. (d) Lanczos (black) and HF (red, blue) transmission coefficient as a function of energy at two different connectivities at $U = U_0$ and $d = d_0$.

a

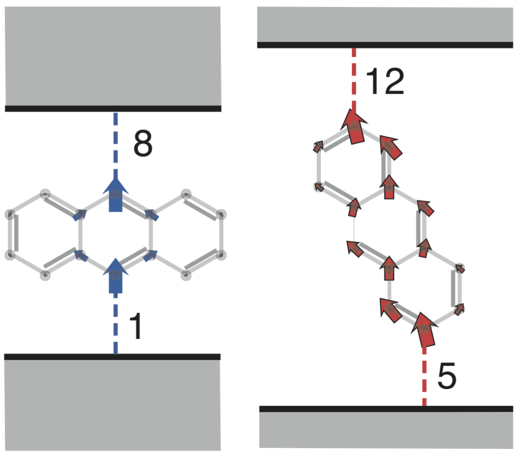

b

λ	ratio HF	ratio Lanczos	gap (eV) HF	gap (eV) Lanczos
0	15.7	15.7	1.99	1.99
0.5	18.7	19.9	4.60	4.51
**1**	**21.2**	**26.3**	**7.29**	**7.18**
1.5	23.7	37.2	10.10	10.21
2	26.6	57.1	13.01	13.77

d/d_0	ratio HF	ratio Lanczos	gap (eV) HF	gap (eV) Lanczos
∞	21.2	26.3	7.29; 7.29	7.18; 7.18
4	21.2	26.3	4.99; 5.61	5.81; 6.07
2	21.6	26.9	5.91; 6.18	4.94; 5.49
1	22.0	27.9	3.88; 4.95	3.88; 4.82

c **d**

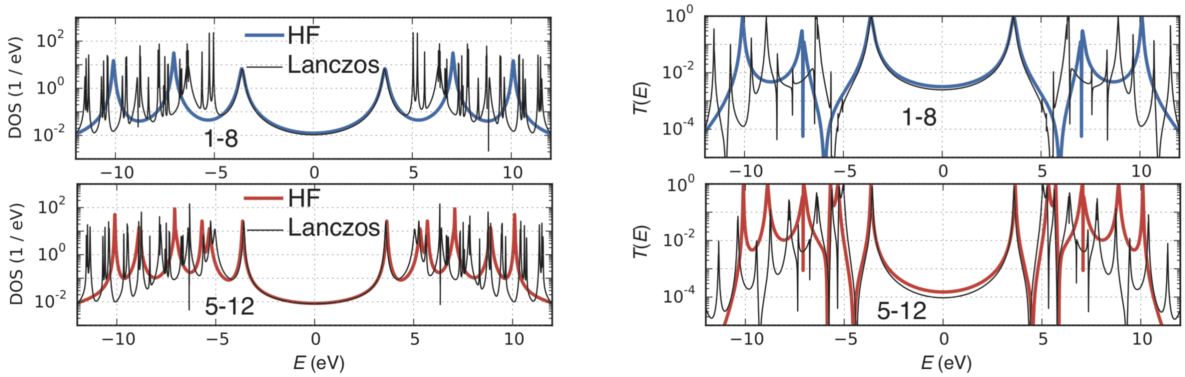

Supplementary Figure 8: results for anthracene
(a) Two examples of anthracene molecule with different connectivities ($1-8$ and $5-12$) to leads (grey area). Arrows represent electron site current. (b) Two tables of conduction ratios and HOMO-LUMO gap according to HF and Lanczos calculation, in the first table for interacting case with no screening with interaction strength $\lambda = U/U_0$ and in the second table for interacting case with screening at interaction strength $U = U_0$. d/d_0 is the distance between lead and connectivity site on the molecule in units of lattice constant. $d = \infty$ corresponds to the case with no screening. The second table shows two different values of HOMO-LUMO gap, where first corresponds to the first connectivity and second to the second connectivity. (c) Lanczos (black) and HF (red, blue) density of states as a function of energy at two different connectivities at $U = U_0$ and $d = d_0$. (d) Lanczos (black) and HF (red, blue) transmitivity as a function of energy at two different connectivities at $U = U_0$ and $d = d_0$.

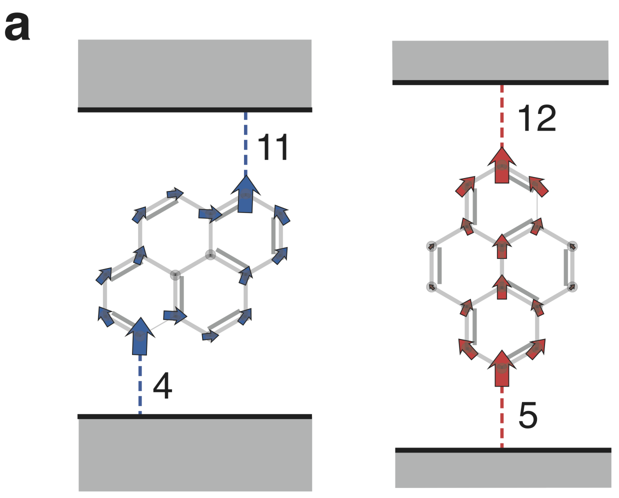

b

λ	ratio HF	gap (eV) HF
0	8.96	2.14
0.5	5.41	4.62
1	**3.92**	**7.26**
1.5	3.01	10.05
2	2.38	12.96

d/d_0	ratio HF	gap (eV) HF
∞	3.92	7.26; 7.26
4	4.42	6.02; 6.16
2	4.92	5.26; 5.61
1	5.78	4.34; 5.03

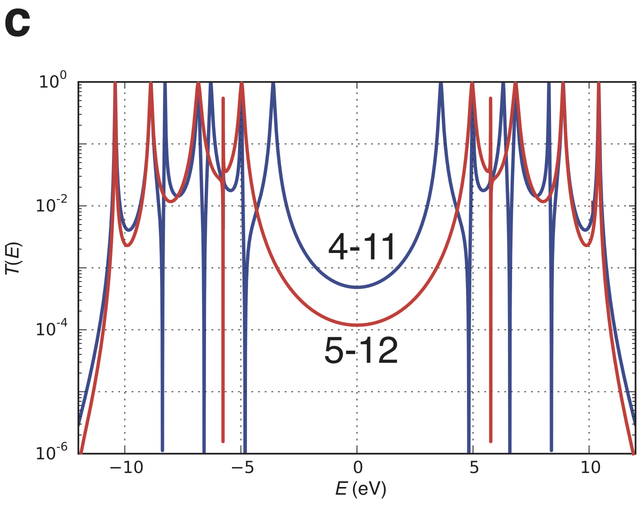

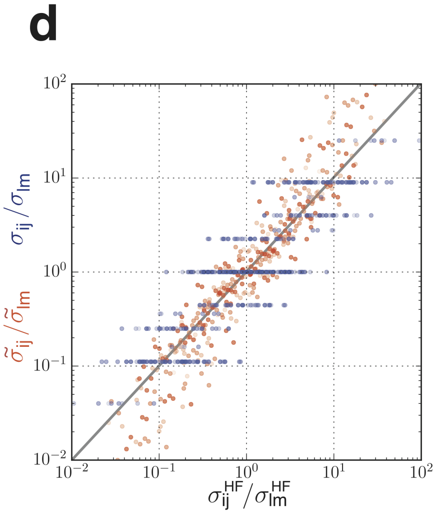

Supplementary Figure 9: results for pyrene
(a) Two examples of pyrene molecule with different connectivities ($4-11$ and $5-12$) to leads (grey area). Arrows represent electron site current. (b) shows two tables of conduction ratios and HOMO-LUMO gap according to HF calculation, in the first table for interacting case with no screening interaction strength $\lambda = U/U_0$ and in the second table for interacting case with screening at interaction strength $U = U_0$. d/d_0 is the distance between lead and connectivity site on the molecule in units of lattice constant. $d = \infty$ corresponds to the case with no screening. The second table shows two different values of HOMO-LUMO gap, where first corresponds to the first connectivity and second to the second connectivity. (c) HF transmitivity as a function of energy at two different connectivities at $U = U_0$ and $d = d_0$. (d) Correlations of the Hartree-Fock conductance ratio (horizontal axis) for a particular pair of connectivities with the non-interacting (blue dots) and the infinite-range interaction (orange dots) conductance ratio for the same pair of connectivities. Results for all possible pairs of connectivities are shown.

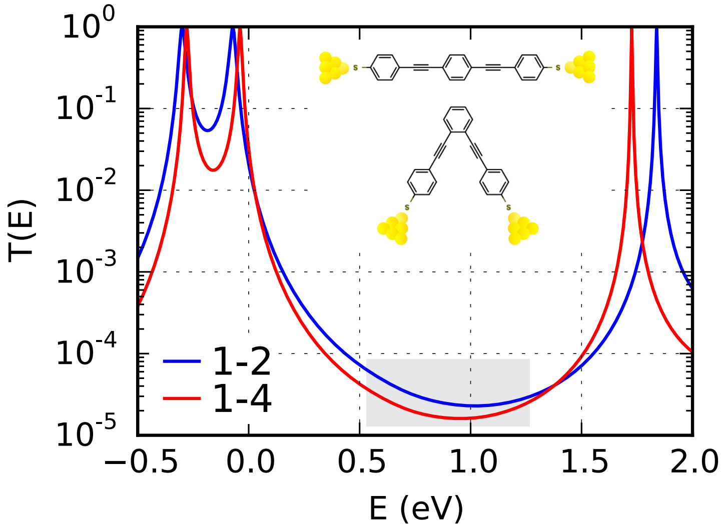

Supplementary Figure 10: DFT results for benzene
DFT results for the transmitivity of benzene with $1-2$ (blue) and $1-4$ (red) connectivities attached to the gold leads. Ratios of conductances in the shaded region of energies approximately coincide with the non-interacting magic ratio rule.

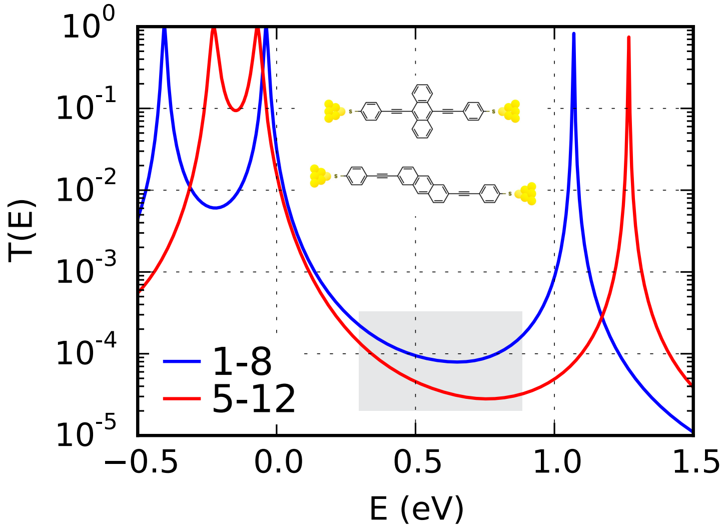

Supplementary Figure 11: DFT results for anthracene
DFT results for the transmitivity of anthracene with $1-8$ (blue) and $5-12$(red) connectivities attached to the gold leads. Ratios of conductances in the shaded region of energies approximately coincide with the non-interacting magic ratio rule.

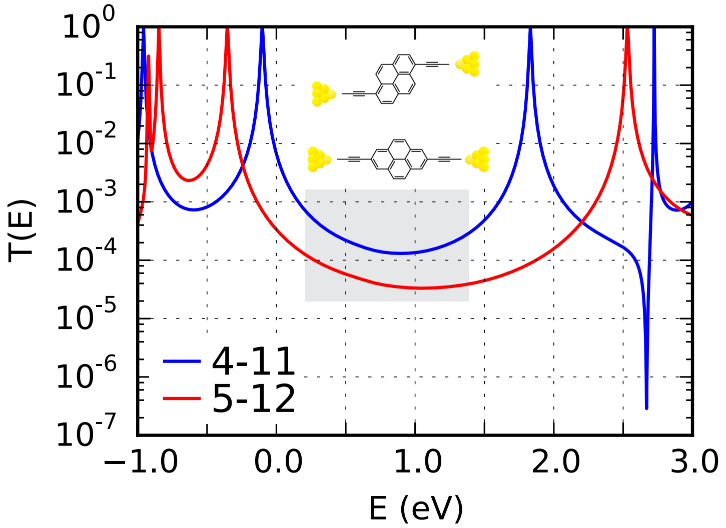

Supplementary Figure 12: DFT results for pyrene
DFT results for the transmitivity of pyrene with $4-11$ (blue) and $5-12$ (red) connectivities attached to the gold leads. Ratios of conductances in the shaded region of energies approximately coincide with the non-interacting magic ratio rule.

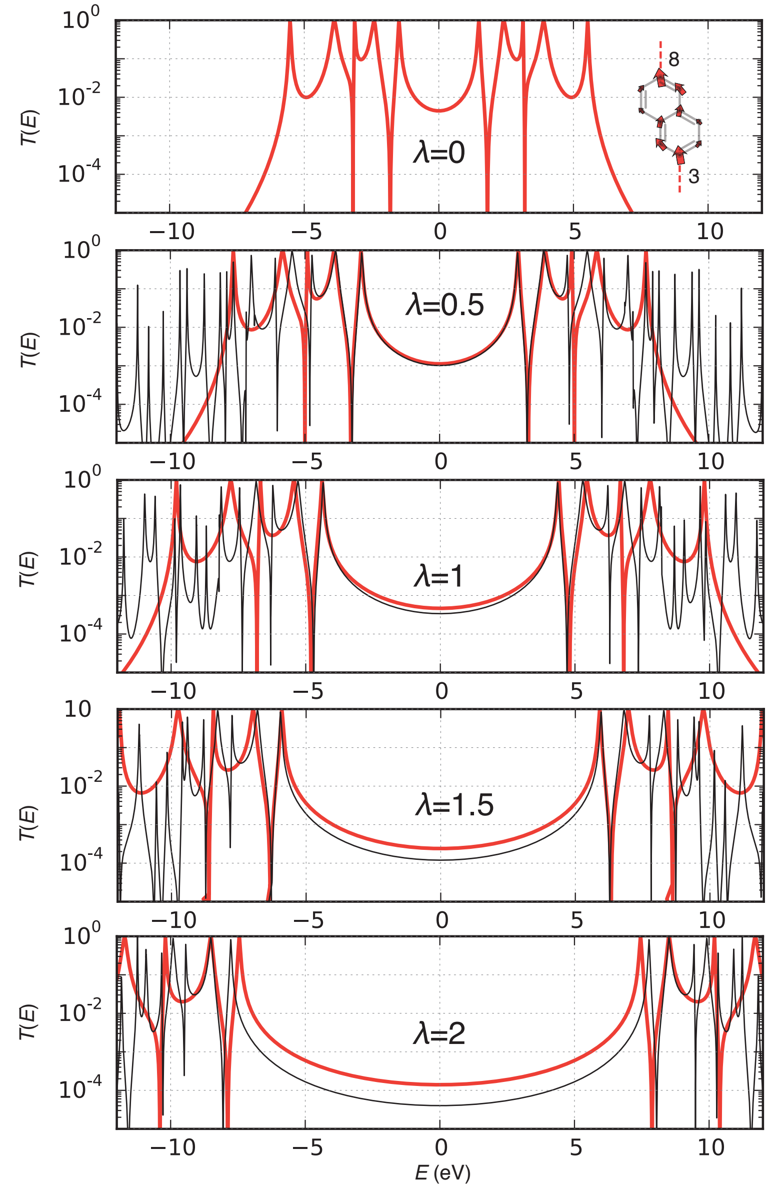

Supplementary Figure 13: Transmitivity of naphthalene at different interactions The comparison of transmitivity of energy from HF (red) and Lanczos (black) calculation for naphthalene connectivity $3-8$ for various interaction strengths $\lambda = U/U_0$ with no screening ($d/d_0 = \infty$).

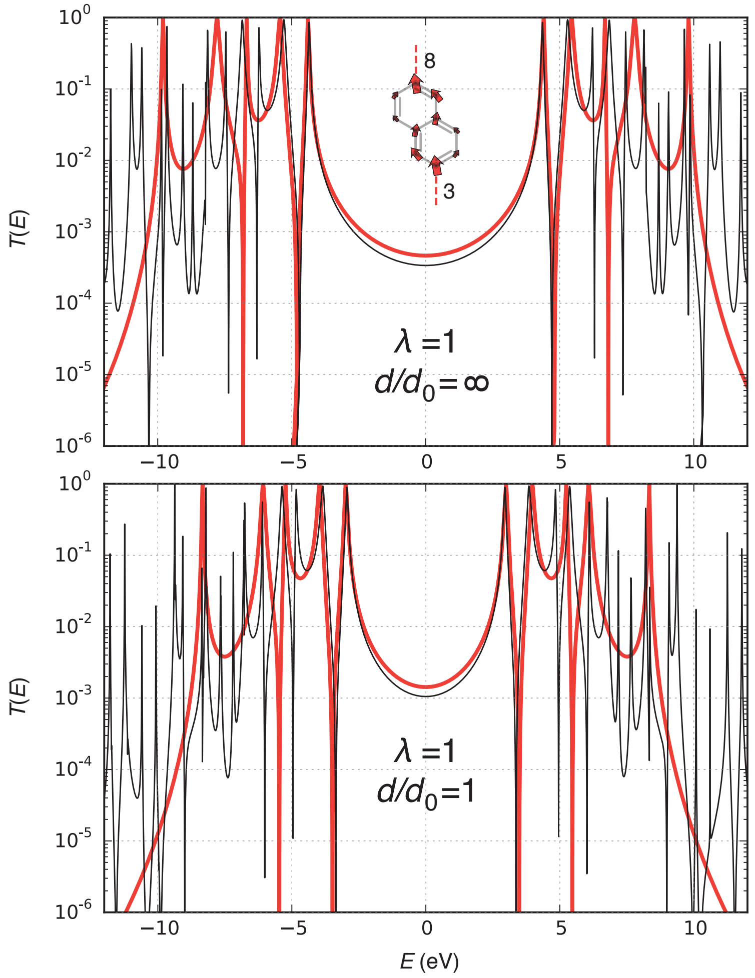

Supplementary Figure 14: Transmitivity of naphthalene with screening
The comparison of transmitivity of energy from HF (red) and Lanczos (black) calculation for naphthalene at connectivity $3-8$ with $\lambda = U/U_0 = 1$ and with screening for different lead distances is shown. d is the distance between the lead and nearest atom on the molecule.

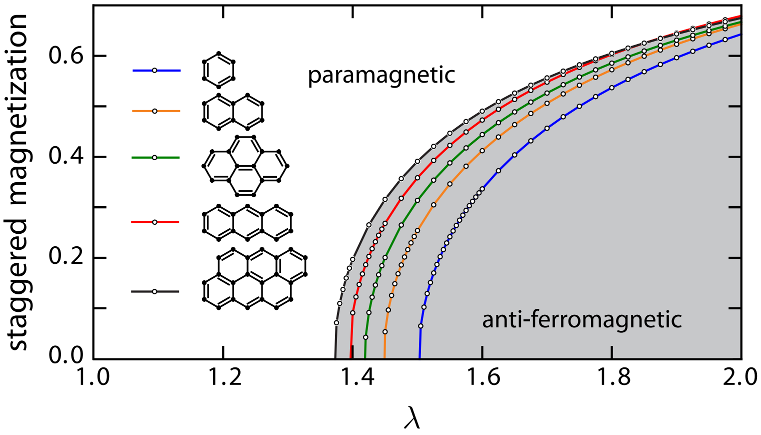

Supplementary Figure 15: Staggered magnetization as a function of interaction strength $\lambda = U/U_0$ for different molecules. Grey shaded area corresponds to an antiferromagnetic phase where $\langle n_{i,\uparrow}\rangle \neq \langle n_{i,\downarrow}\rangle$ and the paramagnetic to the area where the equality holds.

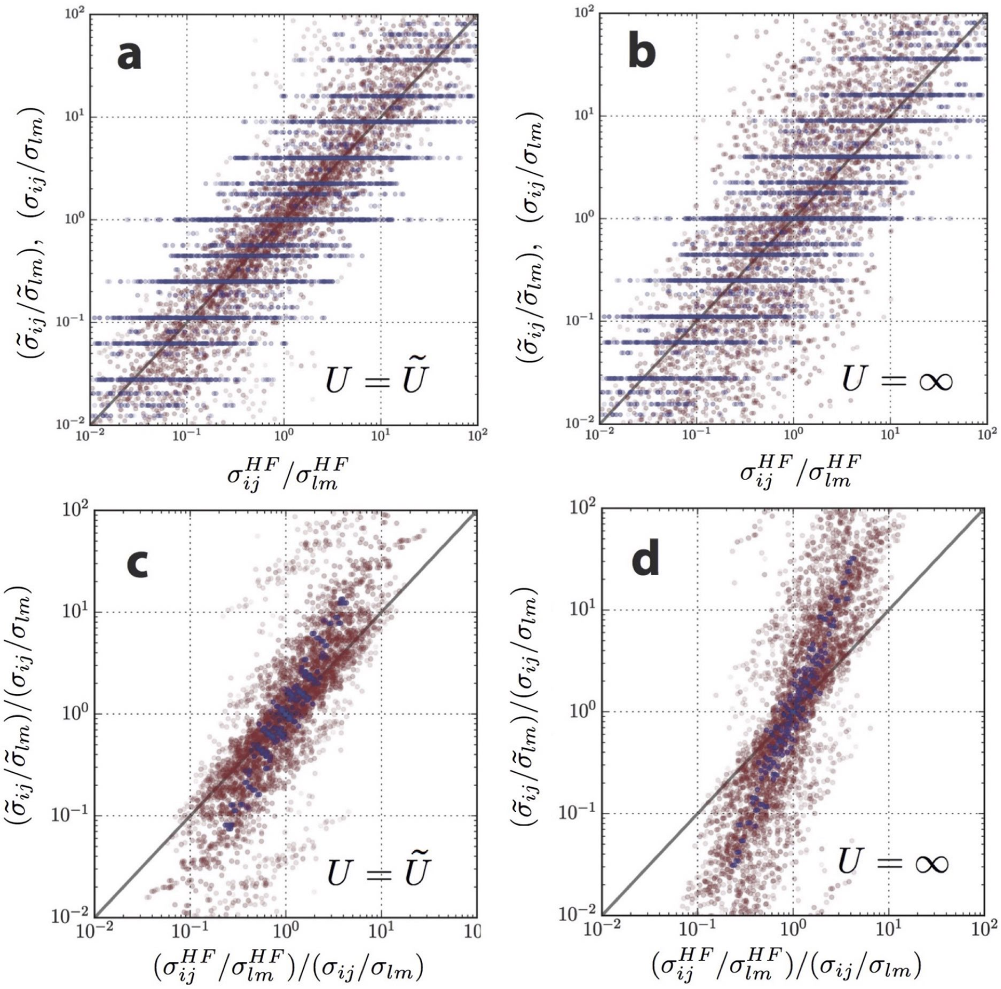

Supplementary Figure 16: results for anthanthrene

(a) Correlations of the Hartree-Fock conductance ratio (horizontal axis, σ_{ij}^{HF}) for a particular pair of connectivities with the non-interacting (blue dots, σ_{ij}) and the infinite-range interaction (orange dots, $\tilde{\sigma}_{ij}$) conductance ratio for the same pair of connectivities (using $U = \tilde{U}$). Results for all possible pairs of connectivities are shown. (b) Similar to (a) but for $U = \infty$. Results for all possible pairs of connectivities are shown. (c) Correlations of the Hartree-Fock conductance ratio (horizontal axis) for a particular pair of connectivities and the infinite-range interaction (orange dots) conductance ratio for the same pair of connectivities and both normalised to the corresponding non-interacting results (using $U = \tilde{U}$). Blue dots represent results for nearest neighbour connectivities only. (d) Similar to (c) but for $U = \infty$.

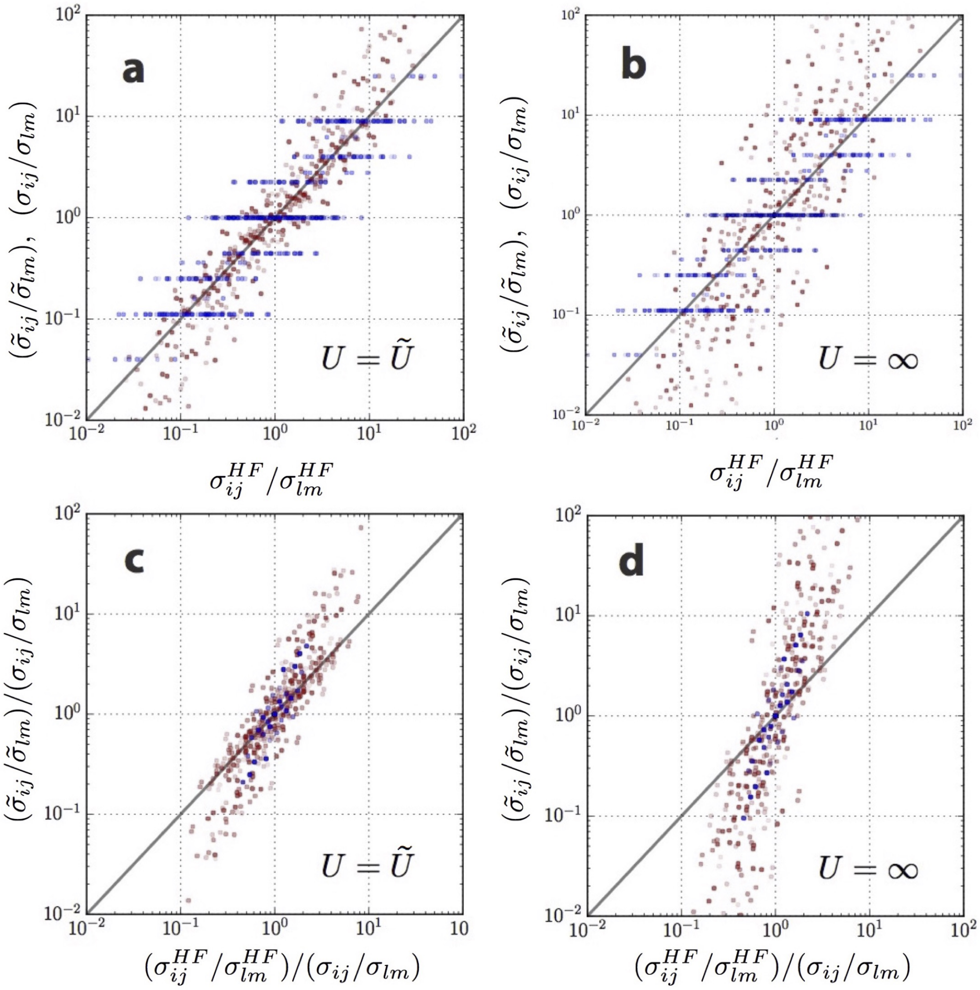

Supplementary Figure 17: results for pyrene
(a) Correlations of the Hartree-Fock conductance ratio (horizontal axis, σ_{ij}^{HF}) for a particular pair of connectivities with the non-interacting (blue dots, σ_{ij}) and the infinite-range interaction (orange dots, $\tilde{\sigma}_{ij}$) conductance ratio for the same pair of connectivities (using $U = \tilde{U}$). Results for all possible pairs of connectivities are shown. (b) Similar to (a) but for $U = \infty$. Results for all possible pairs of connectivities are shown. (c) Correlations of the Hartree-Fock conductance ratio (horizontal axis) for a particular pair of connectivities and the infinite-range interaction (orange dots) conductance ratio for the same pair of connectivities and both normalised to the corresponding non-interacting results (using $U = \tilde{U}$). Blue dots represent results for nearest neighbour connectivities only. (d) Similar to (c) but for $U = \infty$.

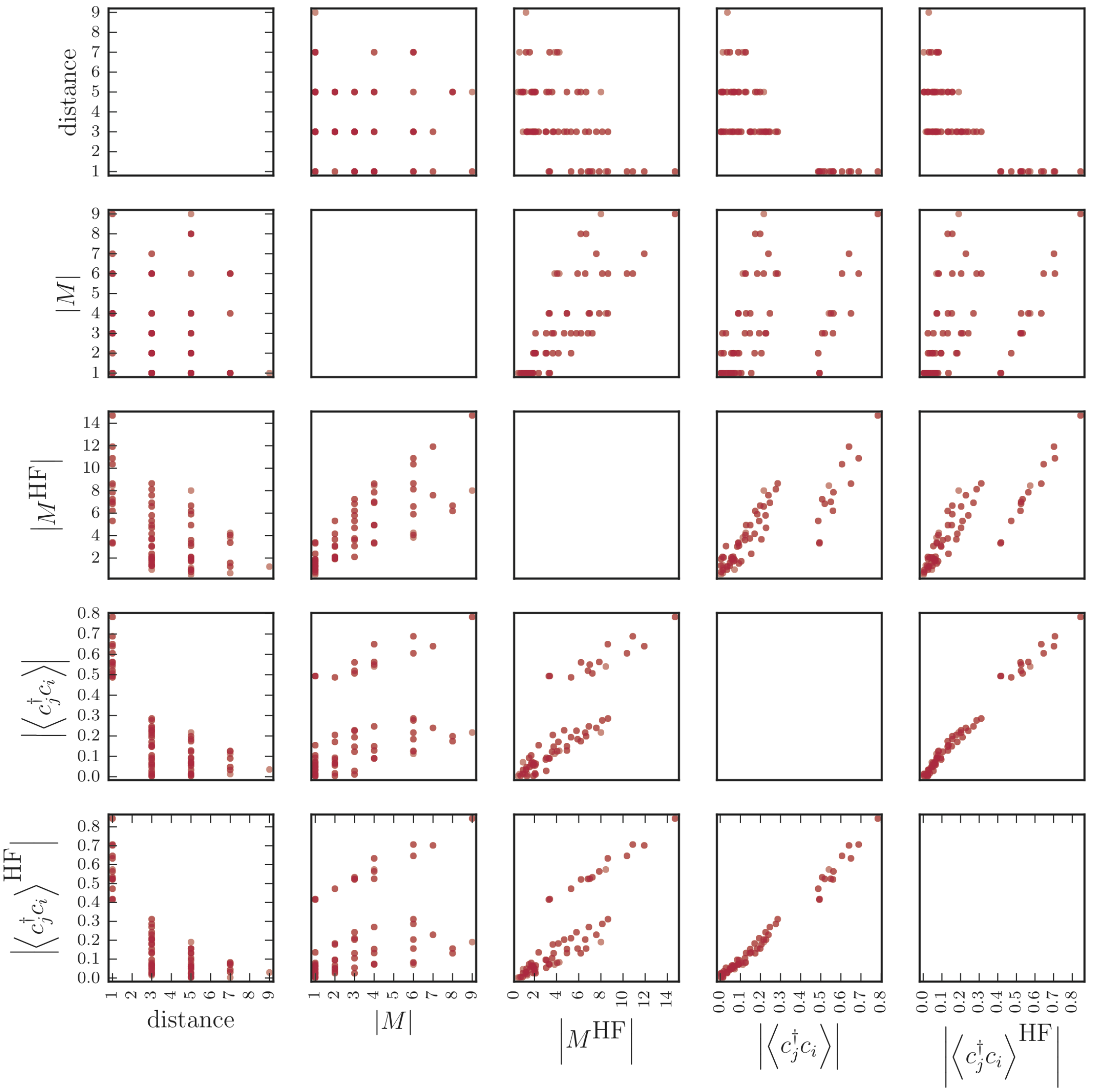

Supplementary Figure 18: correlation graphs for anthanthrene
Correlations of quantities on vertical axes and quantities on horizontal axes in case of anthanthrene. Distance is equal to a number of bonds between site i and site j. Interaction strength is $\lambda = 1$ and no screening is present.

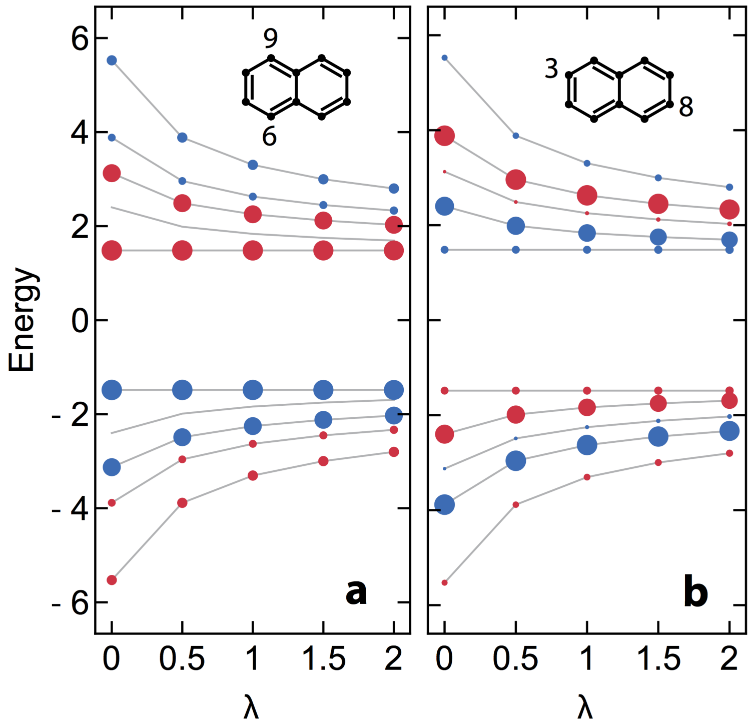

Supplementary Figure 19: Energy levels of naphthalene
Energy levels (grey lines) in naphthalene are shown in dependence to interaction strength λ. They are renormalized so that the HOMO-LUMO gap stays constant. Coloured circles represent $\psi_{k,i}\psi^*_{k,j}$, radius is proportional to magnitude and red/blue colours denote +/− sign.

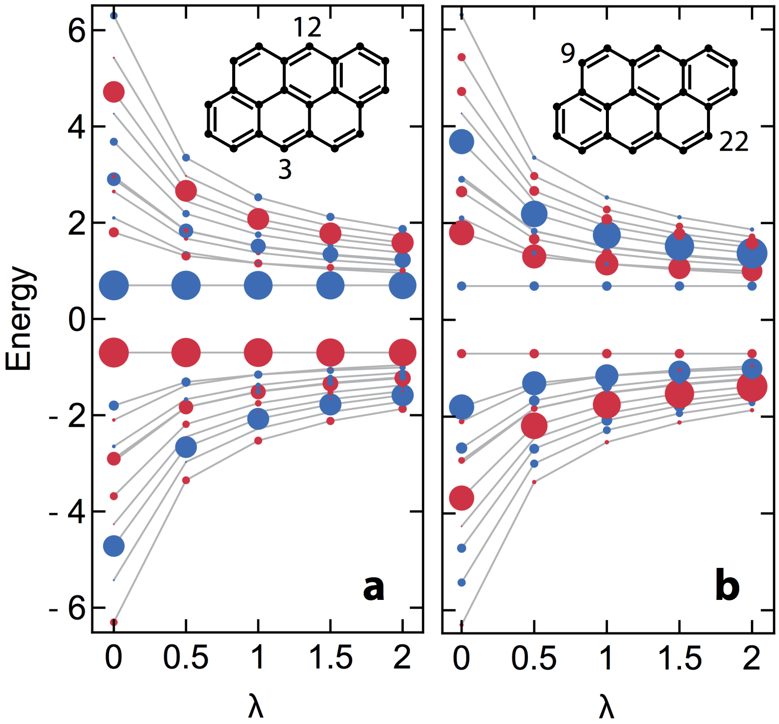

Supplementary Figure 20: Energy levels of anthanthrene
Energy levels (grey lines) in anthanthrene are shown in dependence to interaction strength. They are renormalized so that the HOMO-LUMO gap stays constant. Coloured circles represent $\psi_{k,i}\psi^*_{k,j}$, radius is proportional to magnitude and red/blue colours denote +/− sign.

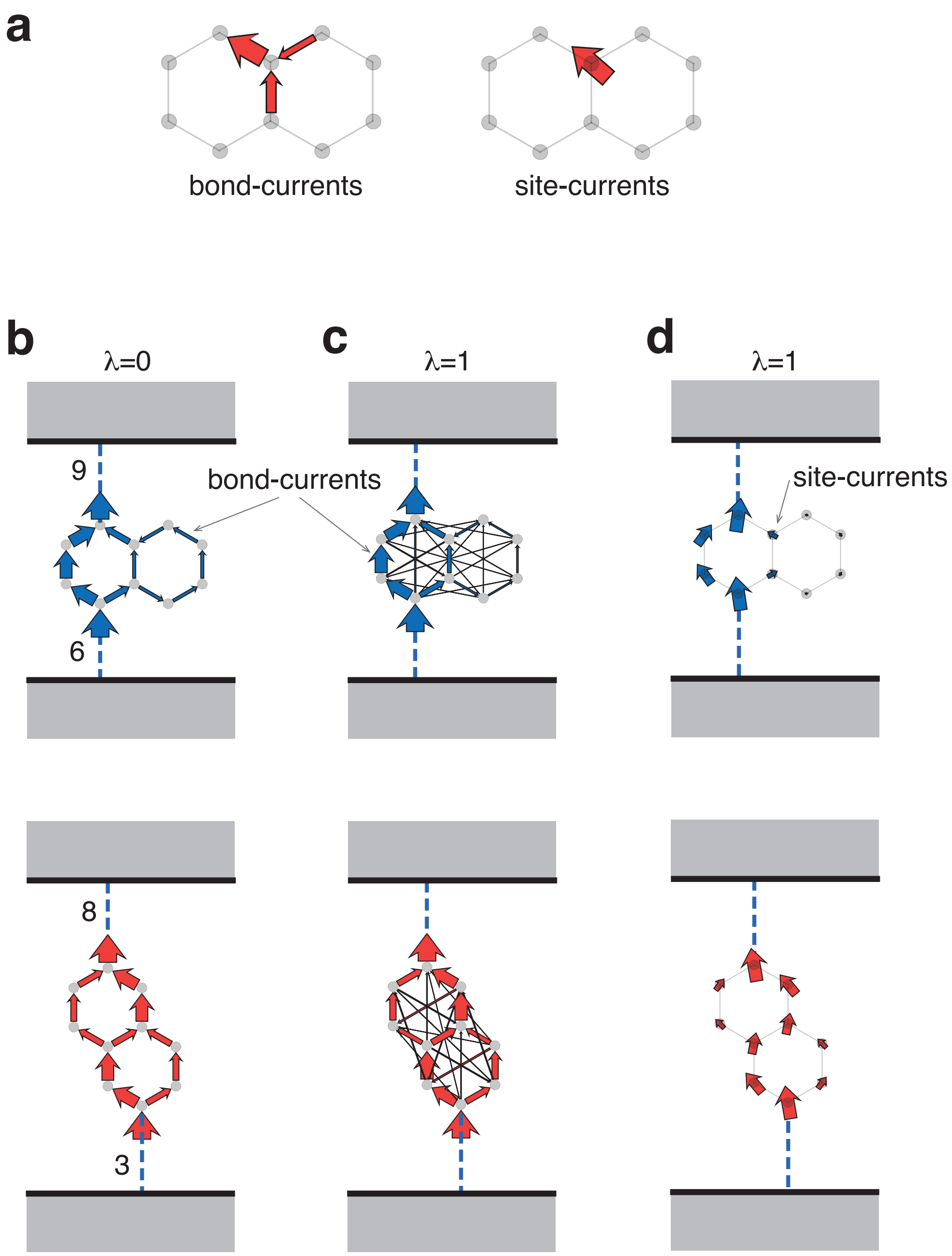

Supplementary Figure 21: Bond and site currents
(a) Bond currents flowing to a particular site of naphthalene and site current, defined as vector sum of bond currents. (b) , (c) and (d) show electron currents in naphthalene connected to leads (grey area) with sites $3-8$ (red) and $6-9$ (blue). In (b) bond currents are shown for a model with no interaction, In (c) are bond current in case of interaction $\lambda = U/U_0 = 1$ and in (d) site currents for system with $\lambda = U/U_0 = 1$.

	distance	$\lvert M\rvert$	$\lvert M^{HF}\rvert$	$\lvert\langle c_j^\dagger c_i\rangle\rvert$	$\lvert\langle c_j^\dagger c_i\rangle^{HF}\rvert$
distance	1.00	-0.12	-0.55	-0.74	-0.76
$\lvert M\rvert$	-0.12	1.00	0.82	0.47	0.48
$\lvert M^{HF}\rvert$	-0.55	0.82	1.00	0.84	0.87
$\lvert\langle c_j^\dagger c_i\rangle\rvert$	-0.74	0.47	0.84	1.00	0.99
$\lvert\langle c_j^\dagger c_i\rangle^{HF}\rvert$	-0.76	0.48	0.87	0.99	1.00

Supplementary Table 1: Correlation coefficients
Correlation coefficients $\mathrm{Corr}(x,y)$ for different pairs of quantities, see vertical and horizontal axes. Interaction strength is $U = U_0$ and no screening is present.

	$\frac{\sigma_{ij}^{HF}}{\sigma_{lm}^{HF}}\rvert_{d\to\infty}$	$\frac{\sigma_{ij}^{HF}}{\sigma_{lm}^{HF}}$	$\left(\frac{[M_{ij}^{HF}]_{ij}}{[M_{lm}^{HF}]_{lm}}\right)^2$	$(\frac{E_g^{lm}}{E_g^{ij}})^2$
Naphthalene $\frac{6-9}{3-8}$	4.41	4.38	3.34	1.30
Anthracene $\frac{1-8}{5-12}$	21.2	22.0	13.5	1.63
Pyrene $\frac{4-11}{5-12}$	3.92	5.78	4.21	1.34
Anthranthrene $\frac{3-12}{9-22}$	148	79.3	60.8	1.31

Supplementary Table 2: Conductance ratios with and without screening
Values of conductance ratios in case of no screening ($d \to \infty$) and screening at distance $d = d_0$ (last three columns), ratio squared of i,j-th element of M-table at connectivity $i - j$ and l,m-th element of M-table at connectivity $l - m$, ratio squared of HOMO-LUMO gap at connectivity $i - j$ and $l - m$. By definition the product of the last two columns should give column 3 but because of non-zero coupling between the molecule and leads, some deviations might occur.

	$\frac{\sigma_{ij}}{\sigma_{lm}}$	$\frac{\sigma_{ij}^{HF}}{\sigma_{lm}^{HF}}$
$3-8/1-6$	1	1.61
$1-8/1-6$	1	1.45
$1-4/1-6$	4	7.16

Supplementary Table 3: Most deviating conduction ratios for naphthalene
Various values of conductance ratios for naphthalene are shown for non-interacting model - giving the magic ratios, and HF calculation of the PPP model with no screening. Although in most cases HF calculations do not deviate significantly from magic ratios, in some cases they do and those are presented in this table. Only connectivities between non-neighbouring sites and sites that have two nearest neighbours are presented since such connectivities are easier to construct in experiment.

$\frac{\sigma_{ij}}{\sigma_{lm}}$	$\frac{\sigma_{ij}^{HF}}{\sigma_{lm}^{HF}}$	
$6-9/1-10$	1	5.06
$6-9/1-12$	1/9	0.66
$15-22/3-16$	1/64	0.0659
$6-9/5-12$	1/64	0.0766
$6-9/5-14$	1/36	0.200
$3-6/5-14$	1/4	0.959
$7-10/5-14$	1	2.98
$6-9/5-16$	1/36	0.178
$15-22/7-16$	1/36	0.163
$15-22/9-22$	1	6.92

Supplementary Table 4: Most deviating conduction ratios for anthanthrene
Various values of conductance ratios for anthanthrene are shown for non-interacting model - giving the magic ratios, and HF calculation of the PPP model with no screening. Although in most cases HF calculations do not deviate significantly from magic ratios, in some cases they do and those are presented in this table. Only connectivities between non-neighbouring sites and sites that have two nearest neighbours are presented since such connectivities are easier to construct in experiment.

References

[1] Pariser, R., Parr, R., G. A semi-empirical theory of the electronic spectra and electronic structure of complex unsaturated molecules. I. *J. Chem. Phys.* **21**, 466-471 (1953).

[2] Pople, J. A. Electron interaction in unsaturated hydrocarbons. *Trans. Faraday Soc.* **49**, 1375-1385 (1953).

[3] Reich, S., Maultzsch, J., Thomsen, C., Ordejn, P. Tight-binding description of graphene. *Phys. Rev. B* **66**, 035412 (2002).

[4] Ohno, K., Some remarks on the Pariser-Parr-Pople method. *Theor. Chim. Acta* **2**, 291-227 (1964).

[5] Perrin, M. L. et al. Large tunable image-charge effects in single-molecule junctions. *Nature Nanotech.* **8**, 282-287 (2013).

[6] Kaasbjerg, K., Flensberg, K. Image charge effects in single-molecule junctions: Breaking of symmetries and negative-differential resistance in a benzene single-electron transistor. *Phys. Rev. B* **84**, 115457 (2011).

[7] Chiappe, G., Louis, E., San-Fabian, E., Verges, J. A. Can model Hamiltonians describe the electron-electron interaction in π-conjugated systems: PAH and graphene. *J. Phys. Condens. Matter* **27** 46, (2015).

[8] Landauer, R. Electrical resistance of disordered one-dimensional lattices. *Philos. Mag.* **21**, 863-867 (1970).

[9] Büttiker, M. Four-Terminal Phase-Coherent Conductance. *Phys. Rev. Lett.* **57**, 1761 (1986).

[10] Averin, D. V., Nazarov, Yu. V. Virtual electron diffusion during quantum tunneling of the electric charge. *Phys. Rev. Lett.* **65**, 2446 (1990).

[11] Groshev, A., Ivanov, T., Valtchinov, V. Charging effects of a single quantum level in a box. *Phys. Rev. Lett.* **66**, 1082 (1991).

[12] Bergfield, J. P., Stafford, C. A. Many-body theory of electronic transport in single-molecule heterojunctions. *Phys. Rev. B* **79**, 245125 (2009).

[13] Geng, Y. et al. Magic ratios for connectivity-driven electrical conductance of graphene-like molecules. *J. Am. Chem. Soc.* **137**, 4469-4476 (2015).

[14] Mahan, G. D. *Many-Particle Physics*, Springer (2000).

[15] Schüler, M., Rösner, M., Wehling, T. O., Lichtenstein, A. I., Katsnelson, M. I. Optimal Hubbard models for materials with nonlocal coulomb interactions: graphene, silicene, and benzene. *Phys. Rev. Lett.* **111**, 036601 (2013).

[16] Schwabl, F. *Advanced Quantum Mechanics* 4th Edition, Springer (2008), pp 35

[17] Chiu, C., Teo, J. C. Y., Schnyder, A. P., Ryu, S. Classification of topological quantum matter with symmetries. *Rev. Mod. Phys.* **88**, 035005-1 (2016).
